# The effect of pressing needle therapy on depression, anxiety, and sleep for patients in convalescence from COVID-19

**DOI:** 10.3389/fneur.2024.1481557

**Published:** 2024-12-18

**Authors:** Ruilong Liang, Lin Tang, Lutong Li, Na Zhao, Xintong Yu, Jinjin Li, Qianqian Wang, Haifeng Cun, Xiaolin Gao, Wenjia Yang

**Affiliations:** ^1^Department of Acupuncture and Moxibustion, Yueyang Hospital of Integrated Traditional Chinese and Western Medicine, Shanghai University of Traditional Chinese Medicine, Shanghai, China; ^2^Department of Rehabilitation Medicine, Yueyang Hospital of Integrated Traditional Chinese and Western Medicine, Shanghai University of Traditional Chinese Medicine, Shanghai, China; ^3^Acupuncture Anesthesia Clinical Research Institute, Yueyang Hospital of Integrated Traditional Chinese and Western Medicine, Shanghai University of Traditional Chinese Medicine, Shanghai, China; ^4^Department of Rehabilitation Medicine, Shanghai Fourth People's Hospital Affiliated Tongji University, Shanghai, China

**Keywords:** pressing needle therapy, COVID-19, depression, anxiety, sleep

## Abstract

**Objective:**

To evaluate the effect of pressing needle therapy on depression, anxiety, and sleep in patients recovering from COVID-19, and to provide a more effective and convenient treatment for the sequelae of COVID-19.

**Methods:**

A total of 136 patients recovering from COVID-19 were randomized into a treatment group (68 cases) and a control group (68 cases, with one case dropping out). The treatment group received pressing needle therapy, while the control group received sham pressing needle therapy, three times a week for 4 weeks. The Patient Health Questionnaire (PHQ-9), Generalized Anxiety Disorder Scale (GAD-7), and Insomnia Severity Index (ISI) were used to evaluate patients’ emotional states and sleep quality. These scales were assessed before, after, and at a 1-month follow-up.

**Results:**

Compared to before treatment, the treatment group showed a significant decrease in PHQ-9 scores (*p* < 0.05, Cohen’s d = 1.26), GAD-7 scores (*p* < 0.05, Cohen’s d = 1.10), and ISI scores (*p* < 0.05, Cohen’s d = 0.94) after treatment. Similarly, at the 1-month follow-up, significant decreases were observed in PHQ-9 scores (*p* < 0.05, Cohen’s d = 1.11), GAD-7 scores (*p* < 0.05, Cohen’s d = 0.88), and ISI scores (*p* < 0.05, Cohen’s d = 0.94). In contrast, the control group demonstrated no statistically significant differences in PHQ-9, GAD-7, or ISI scores after treatment or at the 1-month follow-up (*p* > 0.05). Between the two groups, statistically significant improvements (*p* < 0.05) were observed in PHQ-9 scores (Cohen’s d = 1.47), GAD-7 scores (Cohen’s d = 1.61), and ISI scores (Cohen’s d = 1.06) after treatment. At the 1-month follow-up, statistically significant differences (*p* < 0.05) between the two groups were also noted in PHQ-9 scores (Cohen’s d = 1.10), GAD-7 scores (Cohen’s d = 0.87), and ISI scores (Cohen’s d = 0.92).

**Conclusion:**

Pressing needle therapy significantly improves the mental health and sleep quality of patients recovering from COVID-19. It enhances their quality of life, promotes early recovery, and is simple and easy to administer, making it a treatment worthy of clinical application.

**Clinical trial registration:**

https://www.chictr.org.cn/.

## Introduction

Coronavirus disease 2019 (COVID-19) is an acute respiratory infectious disease that is characterized by occult onset, high infectivity, rapid transmission, general susceptibility of the population, interpersonal transmission, and aggregation (1–4). Since December 2019, more than 200 countries and regions have reported confirmed cases of COVID-19, causing global panic. COVID-19 is highly infectious, spreads rapidly, and causes heavy damage to public health. As the number of infections and deaths has been increasing over time, people have endured severe physical and psychological suffering. In addition to the general population, certain workers are more exposed to COVID-19, particularly healthcare professionals such as medics. Studies pointed out the increased risk faced by these workers due to their direct contact with infected individuals and the nature of their work in medical settings (5) and the rate of COVID-19 was high in healthcare workers (6). Vaccination has been a critical tool in combating the pandemic, the World Health Organization emphasizes the importance of vaccination, stating that safe and effective vaccines help ensure that COVID-19 does not lead to severe outcomes, especially for the elderly, those with chronic diseases, and immunocompromised individuals or pregnant women (7). Regarding diagnosis, various methods have been employed to detect SARS-CoV-2, including real-time reverse transcription–polymerase chain reaction and antigen testing (8). For treatment, a range of strategies has been used, from supportive care to the use of antiviral medications and hydroxychloroquine, the management of COVID-19 patients also frequently includes oxygen therapy (9).

It has been shown that the COVID-19 epidemic causes severe psychological stress to the public, causing panic, anxiety, and other psychological reactions. Once infected, patients are prone to experiencing anxiety, fear, sleep disorders, acute stress disorder, and other pathological psychological events (1–3). An international collaborative study that examined incident anxiety and depression in 22,330 adults from 13 countries on four continents during the COVID-19 pandemic revealed that 25.6% of the population had anxiety and 23.1% of them had depression. These findings suggest that anxiety and depression are prevalent during the COVID-19 pandemic, and public health prevention programs are needed to prevent depression and anxiety and reduce the long-term adverse outcomes associated with depression, anxiety, and mental health problems (4). The incidence of depression, anxiety, and insomnia is higher in infected patients. The results of a quality meta-analysis showed that the combined prevalence of depression, anxiety, and insomnia symptoms was 38, 38, and 48%, respectively, indicating that the proportion of depression, anxiety, and insomnia symptoms in COVID-19 patients was very high (10). In addition, symptoms such as anxiety, depression, and insomnia can persist during the recovery period of COVID-19 (11). The results of a 1-month follow-up of COVID-19 patients showed that psychological symptoms were widespread, including anxiety (42%), insomnia (40%), depression (31%), post-traumatic stress disorder (PTSD) (28%), and obsessive-compulsive symptoms (20%) (12). The latest follow-up report has shown that in some patients the psychological symptoms can last up to 16 months after discharge (13). Therefore, the mental health assessment of COVID-19 patients in the recovery period needs to be carried out for a long time, and effective and safe intervention measures should be actively taken to relieve the mental stress of the recovery population and improve their quality of life.

Traditional Chinese medicine (TCM) has a long history and a significant effect on the treatment of acute infectious diseases, especially viral infectious diseases (14). TCM treatment has been included in the Diagnosis and Treatment Plan for Novel Coronavirus Pneumonia (Trial 7th edition), which was jointly issued by the National Health Commission and the State Administration of Traditional Chinese Medicine. Acupuncture and moxibustion have been actively involved in the prevention and treatment of the novel coronavirus pneumonia and have achieved good results.

Acupuncture and moxibustion are an important part of TCM, with their own distinct characteristics and unique advantages and have made important contributions to the history of China’s antiepidemic measures. In 2020, the Chinese Acupuncture-Moxibustion Society issued the “Guidelines on Acupuncture-Moxibustion Intervention for COVID-19 (the First Edition) (the Second Edition)” (15, 16), which clearly guided the application of acupuncture–moxibustion in COVID-19. During the epidemic of COVID-19 in 2020, Yueyang Hospital of Integrated Traditional Chinese and Western Medicine Affiliated to Shanghai University of Traditional Chinese Medicine used acupuncture to treat COVID-19, and the curative effect was significant (17). On March 15, 2022, the National Health and Wellness Commission and the State Administration of Traditional Chinese Medicine issued the Diagnosis and Treatment Plan for COVID-19 (Trial Ninth Edition) (18), which added the content of “acupuncture and moxibustion treatment” and further clarified the role of acupuncture and moxibustion in the prevention and treatment of COVID-19. In the process of treating patients in shelter hospital or designated hospital for patients affected by COVID-19, we found that acupuncture effectively relieved the clinical symptoms. But treating COVID-19 patients with acupuncture by doctors wearing protective suits is inconvenient and carries certain risks. Therefore, we use pressure acupuncture therapy. A study has reported that pressing needle can improve the clinical symptoms of COVID-19 patients (19) and the use of pressing needle to regulate mental problems is feasible and effective. The operation of pressing needle is simple and feasible, is not limited by the site, and is easy to be adopted by the patients. Pressing needle has been shown to alleviate depression by inducing alterations in vagal function (20). Furthermore, scholars posit that pressing needle may exert its effects by activating the neuro-endocrine-immune network. Research indicates that the skin possesses a complete neuro-endocrine-immune network (21). When pressing needle is inserted into the skin or when the acupoints are intermittently pressed after insertion, the networks are activated one by one and play a combined role to enhance the curative effect of acupuncture (22), and has been shown have positive effects on anxiety, depression and insomnia (23). This activation includes the modulation of serotonin (5-HT) release and regulation in skin (24), collateral vessels (25), nerve terminals and local neurotransmitters (23),as well as the regulation of various immune responses (26). Additionally, animal studies have revealed that hypothalamic–pituitary–adrenal (HPA) axis and the rhythmic secretion of cortisol (27). However, there have been no reports on the use of pressing needle to improve depression, anxiety, and insomnia in patients in convalescence from COVID-19.

A randomized, single-blind, placebo-controlled clinical design was used in this study, to investigate the effects of pressing needles in COVID-19 convalescent patients. We observed improvements in depression, anxiety, and insomnia, providing a more effective and convenient therapy method for COVID-19 convalescence patients.

## Methods

### Study design

**Figure 1:**
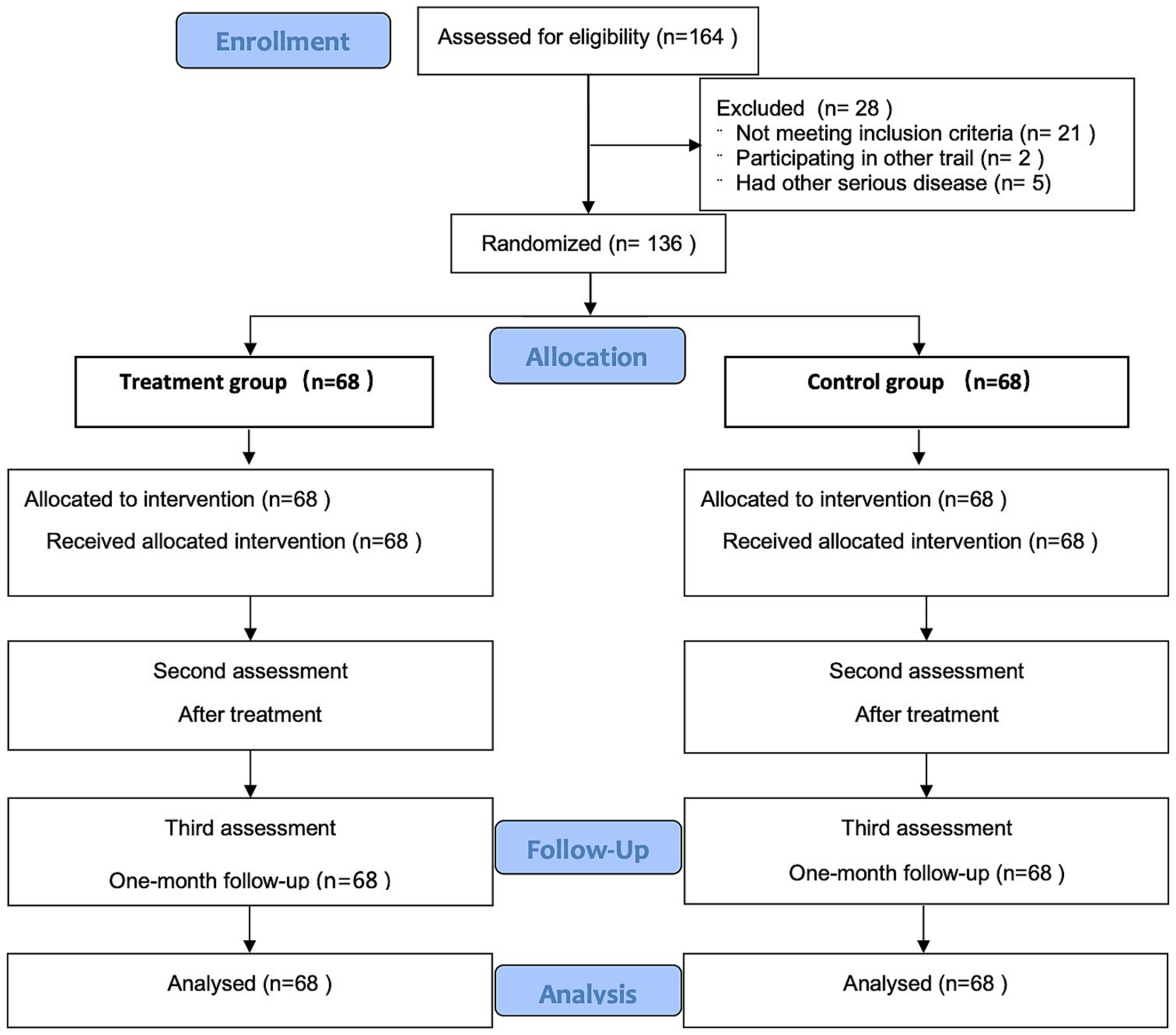
Study flowchart.

This was a patient-blinded, randomized, and placebo-controlled trial aimed at evaluating the efficacy of pressing needle therapy on depression, anxiety, and sleep for patients in convalescence from COVID-19. The trial was conducted at Yueyang Hospital of Integrated Traditional Chinese and Western Medicine affiliated with Shanghai University of Traditional Chinese Medicine. The detailed trial process is presented in Figure 1. This study adhered to the Consolidated Standards of Reporting Trials (CONSORT) (28) and Standards for Reporting Interventions in Clinical Trials of Acupuncture (STRICTA) (29) guidelines for design and reporting of controlled trials. This study was approved by the Ethics Committee of Yueyang Integrated Hospital of Traditional Chinese and Western Medicine affiliated with Shanghai University of Traditional Chinese Medicine (approval number: 2022-103). Trial registration number: ChiCTR2300069602.

Data were collected at three time points: before treatment (1 week before the first treatment), after the treatment (at the end of the treatment), and at 1-month follow-up. The data included the Patient Health Questionnaire (PHQ-9), the Generalized Anxiety Disorder Scale (GAD-7), and the Insomnia Severity Index (ISI). Data entry and management were completed by a data manager, and data records had to be timely, accurate, complete, and standardized. The data manager used software for data entry and management, and the research process was strictly confidential.

### Participants

The trial was conducted at Yueyang Hospital of Integrated Traditional Chinese and Western Medicine affiliated with Shanghai University of Traditional Chinese Medicine. The trial focused on patients during the convalescent period from COVID-19. Participants were recruited from the Department of Acupuncture and Moxibustion of Yueyang Integrated Hospital of Traditional Chinese and Western Medicine affiliated with Shanghai University of Traditional Chinese Medicine, between December 2022 and February 2023.

The diagnostic criteria for confirmed COVID-19 cases were based on the *Protocol for the Diagnosis and Treatment of Novel Coronavirus Pneumonia (9th edition) issued by the National Health Commission* (18). A positive nucleic acid test for the novel coronavirus was the primary criterion. Additionally, the detection of the specific antibody against the new coronavirus could be used as a reference for the diagnosis in the patients who had not received the new coronavirus vaccine. Covid-19 patients were categorized into two types: patients with mild clinical symptoms and no pneumonia can be defined as the mild type, and patients with fever, dry cough, fatigue, or other symptoms and with pneumonia (identified via imaging) can be defined as the normal type.

The inclusion criteria were as follows: (1) patients with a negative detection of the nucleic acid following a previous positive result, meeting the criteria of mild and common type of diagnosis and treatment of new coronavirus pneumonia (trial version 9); (2) patients aged ≥18 years old and ≤ 80 years old, without gender restrictions; (3) no significant abnormality or body disease before the pandemic; (4) the score of PHQ-9 ≥ 5, the score of GAD-7 ≥ 5, and the score of ISI ≥8; (5) patients who have been taking the same antidepressant or sedative and hypnotic drugs steadily for ≥3 months, or those who have not taken antidepressant or sedative and hypnotic drugs; (6) patients with no prior experience of pressing needle treatment; and (7) patients voluntarily participated in the study and signed an informed consent form.

The exclusion criteria were as follows: (1) other causes of pneumonia such as infection with *Epstein–Barr virus, Mycoplasma pneumoniae*, *Chlamydia pneumonia*, *Mycobacterium tuberculosis*, and respiratory tract *Legionella pneumophila*; (2) patients with chronic respiratory tract disease, bacterial respiratory infection including cough variant asthma, asthma, chronic obstructive pulmonary disease (COPD), bronchiectasis, interstitial pneumonia, organizing pneumonia, bronchitis, otitis media, sinusitis, and tonsillitis; (3) congenital malformation of respiratory tract, abnormal development of lung, vasculitis, and dermatomyositis; (4) Patients with severe pre-existing diseases involving the heart, brain, liver, kidneys, or neuropsychiatric system prior to COVID-19 infection; (5) patients with neuropsychiatric disorders, or with serious diseases that affect survival, such as hematologic diseases, tumors, cachexia, and AIDS; (6) pregnant or lactating women; or (7) patients unsuitable for pressing needle therapy due to conditions such as skin redness, localized skin lesions, or allergies to metal or adhesive tape.

We recruited 164 subjects and excluded 28 of them. Among the 28 excluded subjects, 21 did not meet the diagnostic criteria, 2 had participated in other clinical trials, and 5 had other serious diseases, including 1 case with hemophilia, 2 cases with end-stage renal disease, 1 case with aplastic anemia, and 1 case with COPD. Ultimately, 136 subjects were included in the trial and completed it. The 136 patients were randomly divided into the treatment group and the control group, with 68 cases in each group. Random was carried out through a random-number table. The generation of the random-number table, inclusion, and evaluation were performed independently by different researchers. In the control group, the minimum age was 26 and the maximum age was 74, with an average age of 47.93 ± 14.23 years. Among them, there were 35 males with an average age of 49.74 ± 14.41 years, and 33 females with an average age of 46.00 ± 13.99 years. In the treatment group, the minimum age was 18 and the maximum age was 78, with an average age of 47.07 ± 17.87 years. Among them, there were 40 males with an average age of 46.15 ± 18.32 years, and 28 females with an average age of 48.39 ± 17.44 years. No statistically significant differences in gender or age were found between the two groups.

### Interventions

All acupuncturists that took part in this study were licensed doctors with 5–10 years of experience in acupuncture treatment, and they joined the clinical training before the intervention to ensure the standard real and sham acupuncture operation. The participants in the treatment group and the control group received acupuncture or sham acupuncture treatment. Both groups received 12 sessions of different treatments, three times per week for 4 weeks.

**Figure 2:**
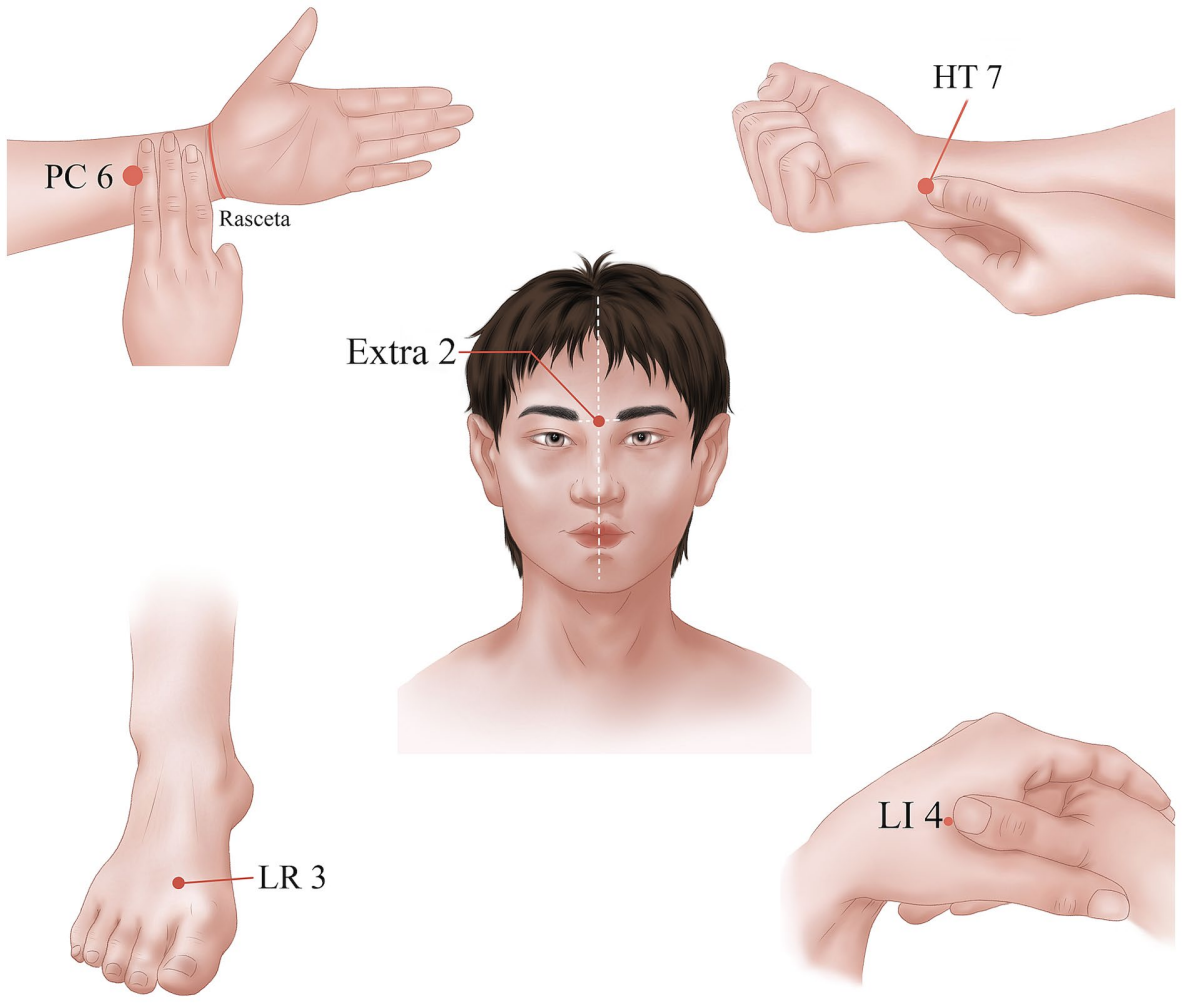
Locations of the acupoints.

**Figure 3:**
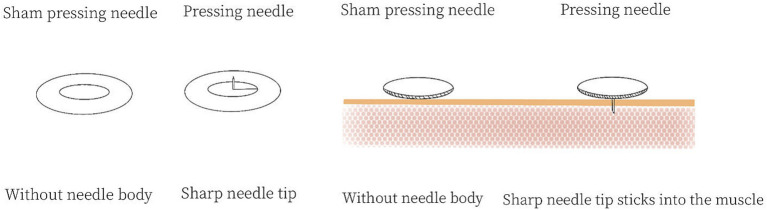
Pressing needle and sham pressing needle.

In the treatment group, the following acupoints were selected: Yintang (Extra 2), bilateral Shenmen (HT7), bilateral Neiguan (PC6), bilateral Hegu (LI 4), and bilateral Taichong (LR3). The acupoints were selected in line with the national standard of People’s Republic of China: Name and Location of acupoints (GB/T12346-2021) (30). The location and acupuncture method of each acupoint are shown in Table 1 and Figure 2. Disposable pressing needles (0.20 × 0.9 mm; Shanghai Youmuran Medical Instrument Co., Ltd.) (shown in Figure 3) were used in this study. Operation: With each patient in a prone or sitting position, the patient’s skin was fully exposed, the skin of the acupoint area was disinfected, and a needle was taken and buried into the skin without tingling. It was advisable to press the acupoint area four times a day, and the needles had to be removed after 24 h. Each patient was treated three times a week for 4 weeks.

**Table 1:** Location and acupuncture method for each acupoint (89).

Acupoint	Location
Yintang (Extra 2)	Midway between the medial ends of the two eyebrows.
Shenmen (HT7)	At the ulnar end of the transverse crease of the wrist, in the depression on the radial side of the tendon of m. flexor carpi ulnaris.
Neiguan (PC6)	2 cun* above the transverse crease of the wrist, between the tendons of m. palmaris longus and m. flexor radialis.
Hegu (LI4)	On the dorsum of the hand, between the 1st and 2nd metacarpal bones, approximately in the middle of the 2nd metacarpal bone on the radial side.
Taichong (LR3)	On the dorsum of the foot, in the depression distal to the junction of the first and second metatarsal bones.

*cun: The width of the interphalangeal joint of the patient’s thumb is considered to be one cun.

The control group received sham pressing needle, and the same acupoints were selected as in the treatment group. Disposable pressing needles (0.20 × 0.9 mm; Shanghai Youmuran Medical Instrument Co., Ltd.) (shown in Figure 3) were used. The needle tips were removed, leaving only a small iron ring in the adhesive tape without needle body. Operation: With each patient in a prone or sitting position, the patient’s skin was fully exposed, the skin of the acupoint area was disinfected, and a needle was taken and buried into the skin without tingling. It was advisable to press the acupoint area four times a day, and the needles had to be removed after 24 h. Each patient was treated three times a week for 4 weeks.

### Measures

1. Patient Health Questionnaire, PHQ-9 (31): the PHQ-9 is an assessment scale from the Diagnostic and Statistical Manual of Mental Disorders (DSM). It is highly sensitive for detecting depression in both clinical practice and research. The reliability and validity of the scale have been widely confirmed (32, 33). It consists of nine items, each scored with 0, 1, 2, or 3. The total score is the sum of the scores of the nine items and reflects the degree of depression. In line with the international scoring standard, the individuals were classified into five groups based on the total scores as follows: 0–4 points, 5–9 points, 10–14 points, 15–19 points, and 20–27 points, corresponding to no depression, mild depression, moderate depression, moderate to severe depression, and severe depression. All the patients were evaluated before treatment, after treatment, and at 1-month follow-up.
2. Generalized Anxiety Disorder Scale, GAD-7 (34): the GAD-7 is commonly used internationally to screen for anxiety. The scale consists of seven items, each scored with 0, 1, 2, or 3. The total score is the sum of the scores of the seven items and reflects the severity of anxiety. In line with the international scoring standard, the individuals were classified into four groups based on the total scores as follows: 0–5 points, 6–9 points, 10–14 points, and 15–21 points, corresponding to no anxiety, mild anxiety, moderate anxiety, and severe anxiety. The validity and utility of the Chinese version of the GAD-7 have been confirmed (35, 36). All of the patients were evaluated before treatment, after treatment, and at 1-month follow-up.
3. Insomnia Severity Index, ISI (37): the ISI is a widely used tool with high reliability and validity (37, 38), mainly used to evaluate the nature and severity of insomnia and its impact on daytime function. It has been proven to be an effective tool for insomnia screening and as a clinical evaluation tool to test the effect of insomnia intervention studies. The scale is simple and easy to complete. It is composed of seven items, where each component represents the degree of severity and is graded as none, mild, moderate, severe, and very severe, with scores of 0, 1, 2, 3, and 4 points, respectively. The sum of the scores of the seven items is the total ISI score, and higher scores indicate more severe insomnia. A total score of 8–14 points was considered subclinical insomnia, and a total score greater than14 points indicated clinically significant insomnia. All the patients were evaluated before treatment, after treatment, and at 1-month follow-up.

### Sample size

The required sample size was estimated based on the change in PHQ-9 scores. According to previous research (39), the PHQ-9 scores of pressing needle therapy group after intervention was 15.29 ± 2.00, and for the control group was 16.36 ± 2.03. This study hypothesized that pressing needle therapy would reduce the PHQ-9 by 1.0 more than control group, and *σ* = 1.7. For this study, we set *α* = 0.05 and 1 − *β* = 0.90, and used the following formula to calculate the sample size in this trial:

n1=n2=2Zα2+Zβ2×σ2μ2−μ12

Calculating the sample size of each group: *n* = 61; considering the loss factor (according to a 10% loss rate), the number of samples in each group is 68 cases, and the total number of samples in the 2 subgroups is 136 cases.

### Randomization and blinding

SPSS26.0 (IBM SPSS Statistics, IBM Corp, Somers, NY, United States) software was used to generate a list of random numbers, and the patients were randomly assigned to the treatment or control groups in a 1:1 ratio, depending on the order in which they signed the informed consent. The random numbers corresponding to each group were recorded. The treatment plans were randomly grouped, and the random numbers corresponding to different treatment plans were recorded. These random numbers and treatment plans were placed into sealed envelopes, which were numbered. Patients then submitted the sealed envelopes to the acupuncturists for intervention. The patients in the treatment group received acupuncture treatment, and the patients in the control group received placebo treatment. After the intervention, clinical data were collected. During the trial, different investigators were responsible for generating the random-number list, recruiting patients, performing acupuncture interventions, measuring outcomes, and analyzing data. Only the acupuncturists knew the allocation. The patients and other researchers were blinded to the assignment.

### Data collection and management

To ensure standardization, the researchers underwent uniform training before the trial, including questionnaire survey, clinical treatment, indicator detection, and data collection and management. Data were collected at baseline (1 week before the first intervention), after the intervention (at the end of the intervention), and at 1-month follow-up. The collected data included PHQ-9, GAD-7, and ISI. Data entry and management were carried out by data managers using specialized software, while statistical analysts perform data analysis. At the end of the study, the collected data were sent to a designated data manager for entry and management. After the establishment of the database, the main researcher and statistical analyst will lock the data, and the locked data will not be modified. Paper-based materials were sealed and stored securely, while online experimental data were encrypted. Access rights were set, and data could only be accessed after identity verification. Additionally, the research team and participants were made aware of the importance of data security and confidentiality.

### Statistical analysis

SPSS 26.0 software was used for statistical analysis. For measurement data that followed a normal distribution and homogeneity of variance, the mean ± SD was used to describe the central tendency and variability. Comparisons between two groups were performed using an independent-sample *t*-test. Comparisons between multiple time points were conducted using repeated-measures analysis of variance (ANOVA). Categorical data were expressed as frequency (*n*) and percentage (%), and comparisons between groups were made using the chi-square test. In this study, a *p*-value of <0.05 was considered statistically significant.

## Results

### Clinical characteristics of subjects

Table 2 presents the clinical characteristics of the subjects. In the control group, there were 57 cases of asymptomatic infection and 11 mild-type infection. In the treatment group, there were 56 cases of asymptomatic infection and 12 mild-type infection. There were no significant differences in clinical type, underlying diseases, ISI scores, GAD-7 scores, and PHQ-9 scores between the two groups (*p* > 0.05) (Table 2).

**Table 2:** Baseline clinical characteristics of subjects.

Variable	Control (*n* = 68)	Treatment (*n* = 68)	χ^2^/t	*p*
Clinical classification				
Asymptomatic infection	57 (83.82%)	56 (82.35%)	0.052	0.819
Mild	11 (16.18%)	12 (17.65%)		
Underlying disease				
Cardiovascular disease (including hypertension)	7	6	0.085	0.771
Chronic lung disease	0	1	1.007	0.316
Diabetes	2	3	0.208	0.649
Chronic liver disease	1	0	1.007	0.316
Kidney disease	2	1	0.341	0.559
Tumor	0	1	1.007	0.316
Cerebrovascular disease	1	0	1.007	0.316
Diseases of immune system	1	0	1.007	0.316
PHQ-9	14.06 ± 5.34	13.96 ± 5.11	0.115	0.909
GAD-7	12.69 ± 5.97	11.85 ± 5.11	0.880	0.381
ISI	14.47 ± 5.55	14.62 ± 5.87	−0.150	0.881

### The effect of pressing needle on PHQ-9 scores

There was no significant difference in PHQ-9 scores between the two groups before treatment (*p* > 0.05). Comparison of PHQ-9 scores for all subjects at different time points showed a significant effect of time (*F* = 133.000, *p* < 0.001), indicating that PHQ-9 scores changed significantly over time. Comparison of PHQ-9 scores between the two groups showed a significant effect of group (*F* = 41.489, *p* < 0.001), indicating that different treatments had differential effects on the patients’ PHQ-9 scores. There was an interaction effect between group and time (*F* = 133.000, *p* < 0.001), indicating that the trend of change in PHQ-9 scores over time differed between the two treatment methods.

**Figure 4:**
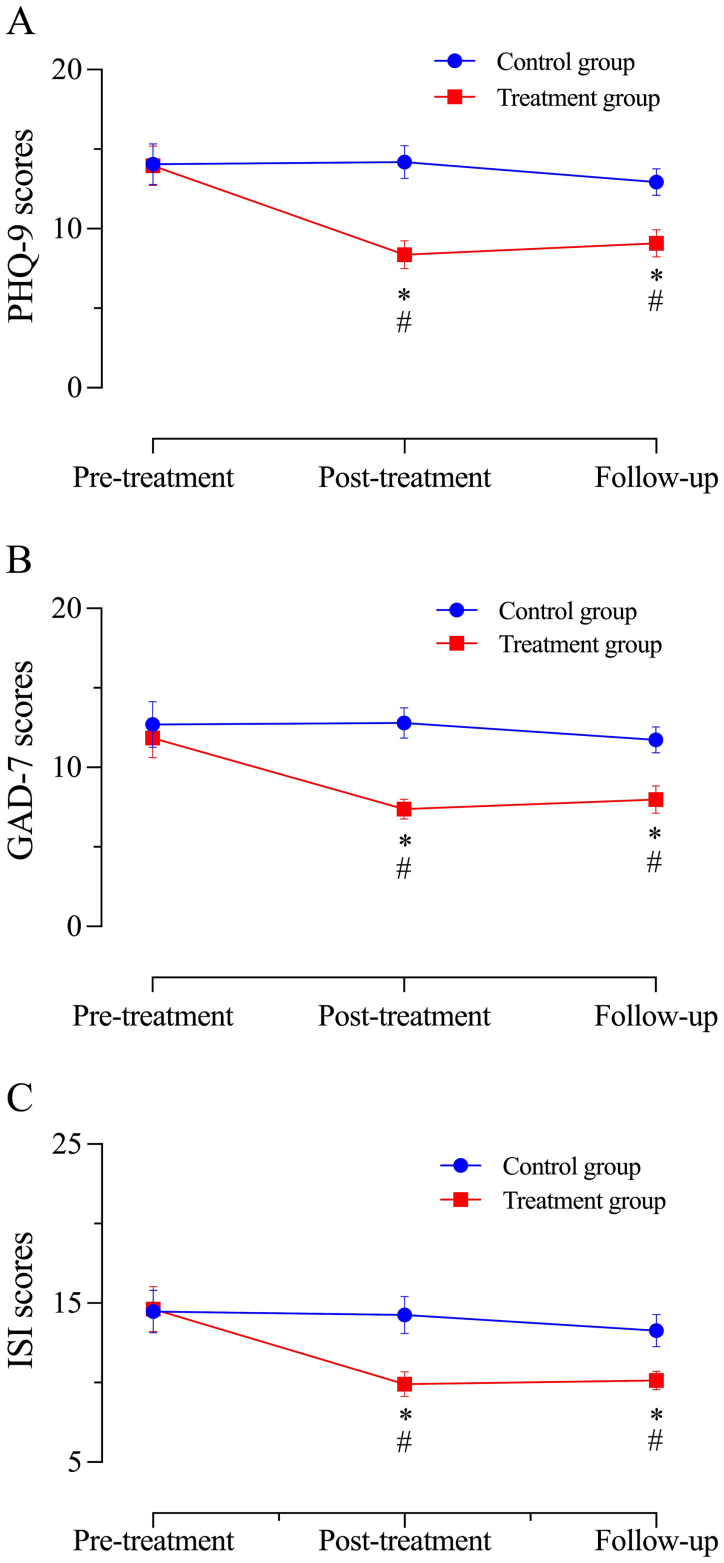
The PHQ-9 scores, GAD-7 scores, and ISI scores of the two groups. **(A–C)** Assessment of PHQ-9 scores, GAD-7 scores, and ISI scores for both groups at pre-treatment, post-treatment, and 1-month follow-up. Means and 95% confidence intervals for: **(A)** PHQ-9 scores; **(B)** GAD-7 scores; and **(C)** SI scores. *: Treatment group vs. control group, *p* < 0.05; #: Compared with before treatment, *p* < 0.05.

Compared to before treatment, the PHQ-9 scores of the treatment group significantly decreased after treatment (*p* < 0.05, Cohen’s d = 1.26), and the PHQ-9 scores of the treatment group also significantly decreased at the 1-month follow-up (*p* < 0.05, Cohen’s d = 1.11). The PHQ-9 scores of the control group after treatment and at 1-month follow-up were not significantly different from those before treatment (*p* > 0.05). After treatment, there was a statistically significant difference in PHQ-9 scores between the treatment group and the control group (*p* < 0.05, Cohen’s d = 1.47). At the 1-month follow-up, there was a statistically significant difference in PHQ-9 scores between the treatment group and the control group (*p* < 0.05, Cohen’s d = 1.10) (Table 3; Figure 4A).

**Table 3:** Comparison of PHQ-9 scores between the two groups.

PHQ-9	Control group (*n* = 68)	Treatment group (*n* = 68)	Effect		
			Time (F/P)	Group (F/P)	Time × Group (F/P)
Pre-treatment	14.06 ± 5.34	13.96 ± 5.11	133.000/<0.001	41.489/<0.001	133.000/<0.001
Post-treatment	14.19 ± 4.29	8.37 ± 3.60*#			
Follow-up	12.93 ± 3.46	9.07 ± 3.56*#			

Data are presented as the mean ± SD. *: Treatment group vs. control group, *p* < 0.05; #: Compared with pre-treatment, *p* < 0.05. PHQ-9, Patient Health Questionnaire.

### The effect of pressing needle on GAD-7 scores

There was no significant difference in GAD-7 scores between the two groups before treatment (*p* > 0.05). Comparison of GAD-7 scores of all subjects at different time points showed an effect of time (*F* = 133.000, *p* < 0.001), indicating that GAD-7 scores changed significantly over time. Comparison of GAD-7 scores between the two groups showed an effect of group (*F* = 50.368, *p* < 0.001), indicating that different treatments can differentially affect GAD-7 scores. There was an interaction effect between group and time (*F* = 133.000, *p* < 0.001), indicating that the change trend of subjects’ GAD-7 scores over time differed with different treatment methods.

Compared to before treatment, the GAD-7 scores of the treatment group significantly decreased after treatment (*p* < 0.05, Cohen’s d = 1.10), and the GAD-7 scores of the treatment group also significantly decreased at the 1-month follow-up (*p* < 0.05, Cohen’s d = 0.88). The GAD-7 scores of the control group after treatment and at 1-month follow-up were not significantly different from those before treatment (*p* > 0.05). After treatment, there was a statistically significant difference in GAD-7 scores between the treatment group and the control group (*p* < 0.05, Cohen’s d = 1.61). At the 1-month follow-up, there was a statistically significant difference in GAD-7 scores between the treatment group and the control group (*p* < 0.05, Cohen’s d = 0.87) (Table 4; Figure 4B).

**Table 4:** Comparison of GAD-7 scores between the two groups.

GAD-7	Control group (*n* = 68)	Treatment group (*n* = 68)	Effect		
			Time (F/P)	Group (F/P)	Time × Group (F/P)
Pre-treatment	12.69 ± 5.97	11.85 ± 5.11	133.000/<0.001	50.368/<0.001	133.000/<0.001
Posttreatment	12.79 ± 3.91	7.38 ± 2.55*#			
Follow-up	11.74 ± 3.34	7.99 ± 3.56*#			

Data are presented as the mean ± SD. *: Treatment group vs. control group, *p* < 0.05; #: Compared with pre-treatment, *p* < 0.05. GAD-7, Generalized Anxiety Disorder Scale.

### The effect of pressing needle on ISI scores

There was no significant difference in ISI scores between the two groups before treatment (*p* > 0.05). Comparison of ISI scores of all subjects at different time points showed an effect of time (*F* = 12.371, *p* < 0.001), indicating that ISI scores changed significantly over time. Comparison of ISI scores between the two groups showed an effect of group (*F* = 8.922, *p* < 0.001), indicating that different treatments can differentially affect the ISI scores. There was an interaction effect between group and time (*F* = 23.504, *p* < 0.001), indicating that the change trend of ISI scores over time was different between the different treatment methods.

Compared to before treatment, the ISI scores of the treatment group significantly decreased after treatment (*p* < 0.05, Cohen’s d = 0.94), and the ISI scores of the treatment group also significantly decreased at the 1-month follow-up (*p* < 0.05, Cohen’s d = 0.94). The ISI scores of the control group after treatment and at 1-month follow-up were not significantly different from those before treatment (*p* > 0.05). After treatment, there was a statistically significant difference in ISI scores between the treatment group and the control group (*p* < 0.05, Cohen’s d = 1.06). At the 1-month follow-up, there was a statistically significant difference in ISI scores between the treatment group and the control group (*p* < 0.05, Cohen’s d = 0.92) (Table 5; Figure 4C).

**Table 5:** Comparison of ISI scores between the two groups.

ISI	Control group (*n* = 68)	Treatment group (*n* = 68)	Effect		
			Time (F/P)	Group (F/P)	Time × Group (F/P)
Pre-treatment	14.35 ± 5.69	14.46 ± 6.06	12.371/<0.001	8.922/<0.001	23.504/<0.001
Posttreatment	14.26 ± 4.83	9.90 ± 3.26*#			
Follow-up	13.26 ± 4.16	10.13 ± 2.38*#			

Data are presented as the mean ± SD. *: Treatment group vs. control group, *p* < 0.05; #: Compared with pre-treatment, *p* < 0.05. ISI, Insomnia Severity Index.

### Safety

In the treatment group, three patients experienced acupuncture-related adverse events, including two cases of skin irritation and one case of pain, resulting in an adverse event incidence of 4.4%. In the control group, one patient experienced acupuncture-related adverse events (skin irritation), with an incidence of 1.4%. No significant difference in the proportion of patients with adverse events was observed between the groups (*p* > 0.05). None of the patients in the trial experienced serious adverse events.

## Discussion

Patients with COVID-19 often report persistent symptoms of anxiety, depression, and insomnia during the recovery period, which can affect their quality of life (11). The prevalence and ongoing uncertainty of COVID-19 have led to an increase in people’s anxiety levels. One study has reported in that due to the fear of the disease and the concerns about spreading the infection to family members and friends, individuals may alter their behaviors (40). For instance, it might lead to excessive precautionary measures such as overusing disinfectants or constantly avoiding public places. Those who were required to reduce social contact and undergo isolation often experience panic, anxiety and depression (41). Anxiety and depression predict the incidence rates of almost all internal medical diseases and somatic symptoms, and increase the risks of most physical health indicators (42). Compared with healthy people, patients with anxiety and depression have significantly lower scores in all dimensions of quality of life (43). Long-term mental and psychological disorders can reduce immunity, prolong the disease, and affect the treatment effect (11), and tragedies such as self-injury and suicide may even occur, leading to long-term negative effects to families and society and affect the development and stability of society. It has been shown that some COVID-19 survivors have a “moderate suicide risk” (44). Lack of sleep is likely to contribute to decreased immunity (45), which is likely to lead to various diseases, such as diabetes (46), heart disease (47), high blood pressure (48), and obesity (49). Sleep quality also has a significant impact on cognitive function and work productivity. Studies have shown that sleep quality, especially in sleep efficiency and sleep fragmentation, and decreased sleep quality has been linked to decreased executive function and increased risk of dementia (50). Decreased sleep quality may also lead to decreased life satisfaction (51). Meanwhile, there is a complex bidirectional relationship among anxiety, depression, and sleep. They influence each other and form a vicious cycle (52). Therefore, it is necessary to carry on the mental health assessment of the convalescent group of COVID-19 patients for a long time and provide effective and safe interventions for these patients as early as possible to relieve their psychological stress and promote their comprehensive rehabilitation (53).

The efficacy of acupuncture in the treatment of COVID-19 patients has been confirmed. A study based on a bioinformatics/network topology strategy was used to explore the targets and molecular mechanisms of acupuncture for COVID-19 and revealing that anti-inflammatory effects, immunity activation, and nervous system modulation were primary therapeutic pathways of acupuncture against COVID-19. Acupuncture may play an active role in the treatment of COVID-19 and deserves further promotion and application (54). Studies have shown that acupuncture can significantly shorten the duration of cough in hospitalized COVID-19 patients (55), it alleviates changes in respiratory signs of moderate to severe COVID patients, including oxygen saturation and respiratory rate (56). Acupuncture can also relieve depression and improve sleep quality of COVID-19 patients, which is probably related to rectifying the imbalanced excitatory and inhibitory neuronal functions (54). As a special acupuncture therapy, pressing needle is simple and effective, easier to be accepted by patients, and suitable for general clinical application. Pressing needle is called the thumbtack-type intradermal needle (57), also known as the screw pin-type intradermal needle. By continuously burying pressing needle in the skin or subcutaneous tissue, a long-lasting and gentle benign stimulation of specific acupoints is obtained, and diseases may be treated by means of its continuous stimulating effect (58). Stimulation of acupoints can improve various long-term symptoms related to COVID-19 (59), pressing needle therapy combines the dual effects of acupressure and acupuncture, studies have shown that acupressure can significantly improve patients’ sleep quality (60, 61) and effectively alleviate anxiety associated with various diseases, making it a potential treatment strategy for long-term COVID-19-related anxiety (62). In the treatment of COVID-19, pressing needle can significantly improve pulmonary ventilation function in patients with COVID-19 during the recovery period (19). Needle pressing therapy can more rapidly improve clinical symptoms in patients with moderate to severe COVID-19, with a faster rate of nucleic acid test conversion to negative and a lower rate of disease progression (63). Some studies have confirmed that pressing needle treatment can improve the symptoms of depression and insomnia in patients (64, 65). Meta-analyses of pressing needle therapy for insomnia has shown that the clinical efficacy of pressing needle therapy is comparable to, or even superior to, traditional acupuncture, the effect of pressing needle therapy in reducing PSQI score is better than that of acupuncture (66, 67). Pressing needle therapy plays a significant role both in standalone applications and in combination with other treatments which is likely due to pressing needle’s ability to provide a continuous and sustained stimulation to acupoints without being limited by time or location.

In this study, we used pressing needle to treat depression, anxiety, and sleep disorders for patients in convalescence from COVID-19. The acupoints we selected included: Yintang (Extra 2), Shenmen (HT7), Neiguan (PC6), Hegu (LI 4), and Taichong (LR3). According to the theory of traditional Chinese medicine, the occurrence of anxiety, depression and insomnia is closely related to the heart, liver and brain. The heart regulates psychological and emotional activities, the liver assists the heart to regulate thinking and emotions, and the brain is closely related to the human spirit, consciousness and thinking. Yintang (Extra 2) is the acupoint of the Du meridian, which is directly related to the brain. In clinical practice, it was observed that acupuncture or acupressure at Yintang (Extra 2) can improve patients’ mental disturbance symptoms (68). Animal experiments and clinical trials also confirmed the anti-anxiety effect of Yintang (Extra 2) (61, 69–71). Shenmen (HT7) is the acupoint of the heart meridian, Neiguan (PC6) is the acupoint of the pericardium meridian, which are both closely related to the heart. Shenmen (HT7) plays an important role in improving anxiety and depression symptoms (72), and can improve cognitive dysfunction caused by insomnia (73). Acupuncture stimulation at Neiguan (PC6) can effectively restore biochemical and behavioral disorders related to chronic mild stress (CMS), and has therapeutic effects on chronic stress-related diseases such as anxiety, depression and insomnia (74). Taichong (LR3) is the acupoint of liver meridian. Taichong (LR3) and Hegu (LI4) are key points to harmonize Qi and blood. Taichong (LR3) is one of the core acupoints for the treatment of depression (75) and Hegu (LI4) can reduce the level of anxiety and depression (76). The combined application of acupoints often has better curative effect than the single point. A clinical trial confirmed that the acupressure of Yintang (Extra 2) and Shenmen (HT7) can reduce stress levels, relieve anxiety and improve sleep quality (77). Transcutaneous acupoint stimulation at Neiguan (PC6), Shenmen (HT7) and Hegu (LI4) can effectively improve sleep quality and reduce inflammatory response in elderly patients after operation (78). Acupuncture at Baihui (GV20), Neiguan (PC6), Shenmen (HT7) and Taichong (LR3) can improve the abnormal behavior of insomnia rats (79). Therefore, Yintang (Extra 2), Shenmen (HT7), Neiguan (PC6), Hegu (LI4) and Taichong (LR3) were selected in our study to improve the mental health and sleep status of patients in convalescence from COVID-19.

We observed the effects of pressing needle therapy on depression, anxiety, and sleep in patients with COVID-19 during the recovery period. PHQ-9, GAD-7, and ISI were used to assess the emotional state and sleep quality of the patients. The PHQ-9 was developed based on the diagnostic criteria for depressive episodes in 2001 and has been used to screen and identify depressive disorders. Due to its good reliability and validity and applicability to people in different language environments, it has been widely used in outpatient clinics of general hospitals in China, demonstrating high reliability and validity (32). The GAD-7 was compiled according to the diagnostic criteria of generalized anxiety disorder in 2006 and has been used to screen for generalized anxiety disorder and evaluate the severity of symptoms. It has good reliability, validity, and repeatability and can be widely used (34, 80, 81). The ISI is the most commonly used measuring method in insomnia research and is regarded as a fundamental measure of sleep and insomnia symptoms worldwide (82, 83). PHQ-9, GAD-7, and ISI are simple, time-efficient, and cost-effective; have good reliability and validity in the identification of depression, anxiety, and insomnia; and are suitable for general use in outpatient clinics. These scales were assessed before treatment, after treatment, and at 1-month follow-up.

The results of this study demonstrate that pressing needle therapy can significantly reduce the scores of PHQ-9, GAD-7, and ISI in patients recovering from COVID-19, alleviate depression, anxiety, and fatigue, and improve sleep quality. This is the first report on the use of pressing needle therapy to address depression, anxiety and sleep in patients recovering from COVID-19. This study confirms that pressing needle therapy has a significant effect on the clinical symptoms of COVID-19 in the recovery period and is simple and easy to operate. It can be adopted as a routine treatment for patients recovering from novel coronavirus infection, as well as for patients with depression, anxiety, and insomnia, so it is worth promoting and applying. Based on previous studies related to acupuncture for emotional problems, we compared our results with theirs. Regarding anxiety, two previous randomized controlled trials reported benefits of electroacupuncture (84, 85). One of these studies highly anxious individuals had their GAD-7 scores reduced to a low level after five treatments, with scores continuing to decrease in subsequent treatments, which is consistent with our results, and in the future we may be able to do studies with larger sample sizes and longer treatment durations to confirm the long-term effects of pressing needle as well as the treatment durations that achieves the optimal results. Three other randomized controlled trials reported the efficacy of acupuncture, with ISI scale scores showing trends similar to our findings (86–88). In addition, we also noted that there were more adverse events of acupuncture in other studies, mainly in the form of headache, localized pain, and subcutaneous hematoma.

In contrast, our study experienced only occasional adverse events, primarily skin irritation.

In conclusion, we believe that pressing needle therapy has similar effects in relieving mood disorders and improving mental health compared with regular acupuncture. Moreover, it is more acceptable and safer, and thus it deserving of extensive promotion and application.

Pressing needle therapy is easy to perform, safe for needling, and well-accepted, with dynamic needle retention and cumulative effects. In hospitals, pressing needle therapy can be integrated into routine treatment and administered by medical staff. In communities, education and training can enable patients and their families to master basic pressing needle techniques, facilitating self-management. Remote guidance can be provided through videos, WeChat, brochures, and other methods to teach patients how to perform pressing needle therapy at home. This makes pressing needle therapy highly suitable for use in modern, busy lifestyles, allowing patients to perform the treatment at home and reduce medical costs. In summary, with its ease of use, safety, and effectiveness, pressing needle therapy can be seamlessly incorporated into mental health and post-COVID recovery plans, making it suitable for a wide range of treatment scenarios.

In drawing our conclusions, we also considered that our research results might be influenced by certain psychosocial factors, such as the fear and threat of death from COVID-19 infection, the stress of adapting to an unfamiliar hospital environment, the emotional strain of separation from loved ones, the guilt of potentially infecting family members, and the use of anti-anxiety, antidepressant, sedative, and hypnotic medications, all of which could affect the efficacy of pressing needle therapy. Additionally, after contracting COVID-19, individuals may experience physical symptoms such as palpitations, difficulty breathing, fatigue, and sweating. These physical symptoms can interact with emotional and sleep issues, thereby affecting the effectiveness of acupressure treatment. Furthermore, the psychological state of COVID-19 patients may change over time, which could also influence the efficacy of acupressure treatment depending on when the cases were collected.

Of course, there are also limitations in this study. First, the sample size is relatively small, and we did not conduct further analysis on potential differences in demographic or clinical characteristics. Secondly, the primary observational indicators of this study are subjective scales, which lack support from objective measures. In future research, we will expand the sample size and include the observation of objective indicators for further analysis. In clinical practice, pressing needle therapy can be combined with other treatment methods, such as psychological care and relaxation training. Depending on the specific circumstances, the duration of needle retention, the number of pressings, and the duration of each pressing can be appropriately adjusted. Optimizing the selection of acupoints and choosing the most suitable points for pressing needle therapy, while also strengthening patient education and self-management by teaching patients how to perform self-pressing and replace needles at home, could improve treatment compliance and continuity. These measures may help enhance the universality and long-term efficacy of pressing needle therapy.

## Data Availability

The raw data supporting the conclusions of this article will be made available by the authors, without undue reservation.
